# Patency of Branch Vessels After Pipeline Embolization: Comparison of Various Branches

**DOI:** 10.3389/fneur.2019.00838

**Published:** 2019-08-07

**Authors:** Xinzhi Wu, Zhongbin Tian, Wenqiang Li, Jian Liu, Yisen Zhang, Ying Zhang, Yangyang Zhou, Xinjian Yang, Shiqing Mu

**Affiliations:** Department of Interventional Neuroradiology, Beijing Neurosurgical Institute, Beijing Tiantan Hospital, Capital Medical University, Beijing, China

**Keywords:** pipeline embolization device, PED, branch, aneurysm, occlusion

## Abstract

**Objective:** Pipeline embolization devices (PEDs) are widely used to exclude intracranial aneurysms from their parent arteries. Side branches covered by PEDs, however, sometimes experience occlusion and related symptoms. Thus, predictors of branch occlusion and the patency and clinical outcomes of these branches are concerning.

**Methods:** We conducted a retrospective review of consecutive patients who had been treated with PEDs in our institution during 2015–2018 to identify those in whom one or more branches were involved. Pretreatment and follow-up angiograms were assessed to determine patency of the involved branches. Demographic and clinical data, treatment strategies, and comorbidities were collected to investigate their relations with branch occlusion.

**Results:** Altogether, 173 branches [24 (13.9%) occluded), 29 (16.8%) with diminished flow] in 126 patients were studied. Five patients (OphA involved) presented with blurred vision and 1 patient (ACA involved) presented with headache. None of the other patients had neurologic complications or clinical strokes related to branch occlusion. Multivariate analysis identified that small PED diameter [*p* = 0.003, odds ratio (OR) = 0.168], branches arising from the aneurysm (*p* = 0.004, OR = 6.614), and involvement of the anterior cerebral artery (ACA) (*p* < 0.001, OR = 25.656) significantly predicted branch occlusion.

**Conclusion:** Branch occlusion rate after PED deployment was low and most occlusions was asymptomatic. Branches with rich collateral supply were more likely to occlude, especially the ACA. Smaller PED diameter, branches arising from the aneurysm, and ACA involvement were significant predictors of branch occlusion after PED treatment.

## Introduction

The pipeline embolization device (PED; Medtronic, Minneapolis, MN, USA) diverts aneurysmal inflow, resulting in stagnant blood flow in the aneurysm—in turn, thrombosis development is promoted, with the construct for neointimal formation a possibility. PED has been shown to exclude aneurysms from their parent arteries (1–3). The rates of complete aneurysmal occlusion treated by PED were reported at 76.0% at 6 months (4) and 95.2% at 5 years (1). Ostium of branch artery were involved after PED deployment sometimes. The patency and clinical outcomes of the involved branches are concerning. Although several studies have focused on the patency of branches, the reported occlusion rates varied greatly (5–7) and the differences in the rates among the various branches were not well defined. We therefore aimed to evaluate the patency and clinical outcomes of side branches after PED deployment, compare the occlusion rates in various branch arteries, and identify the predictors of branch occlusion.

## Materials and Methods

### Study Populations

A retrospective review of consecutive patients treated with the PED in our institution from July 2015 to July 2018 was conducted to identify patients in whom the PED involved one or more branches. Patients who were lost during the follow-up were excluded. Angiograms obtained immediately after the procedure and at follow-up were reviewed to determine the patency of branches and aneurysmal occlusion. Demographic data—patient's age, sex, and clinical data like aneurysm size, treatment strategies, number of PEDs, length of follow-up, comorbidities (e.g., hypertension, coronary artery disease, diabetes mellitus)—were collected to investigate their relations with branch occlusion. Aneurysm size, defined as the maximum aneurysm sac size, was measured using digital subtraction angiography. The angiograms were evaluated by two experienced neurointerventionalists. When disagreements occurred, a third neurointerventionalist not involved in this study became the arbitrator. For patients with more than one follow-up, data from the latest follow-up were used for the investigation. The branch vessels involved in this study included the ophthalmic artery (OphA), posterior communicating artery (PCoA), anterior choroid artery (AChA), anterior cerebral artery (ACA), anterior inferior cerebellar artery (AICA), and posterior inferior cerebellar artery (PICA). Clinical outcomes were investigated after treatment and at follow-up to determine if there were any symptoms related to branch occlusion. The study was approved by the ethics board of Beijing Tiantan Hospital and all patients agreed to participate and signed informed consent forms.

### Procedures

All patients received a dual antiplatelet regimen of aspirin (100 mg/day) and clopidogrel (75 mg/day) for 5 days prior to the intervention. Platelet function was routinely assessed using thromboelastography, with a target platelet inhibition of 30–90%. During the procedure, the patient's activated clotting time was maintained at 250–300 s using intravenous heparin (70 U/kg). All patients were treated under general endotracheal anesthesia. Distal access was obtained using a biaxial or triaxial access system and a Marksman microcatheter. The appropriately sized PED was selected after measuring the parent artery and was deployed to cover the aneurysmal neck. After the procedure, the dual antiplatelet regimen was continued for 6 months and the aspirin for life.

### Patency Assessment

Follow-up was conducted with digital subtraction angiography in all patients. Patency of branch vessels were assessed immediately after the procedure and at follow-up. Blood flow conditions of the involved branch vessels were divided into three types (8): (1) patent—with flow similar to that present before treatment. (2) diminished—patent but with delayed or decreased filling of branches and stenosis at the ostium of the branch; (3) occluded—no filling appears in branches after the procedure.

### Statistical Analysis

Statistical analysis was conducted using SPSS 21.0 software (IBM, Armonk, NY, USA). Data for continuous variables are presented as means and range and for categorical variables as the frequency. Continuous variables were analyzed using unpaired *t*-tests and categorical variables using χ^2^ tests, as appropriate. Significant factors (*p* < 0.05) found in the univariate analysis were then analyzed using a binary logistic regression analysis. Statistical significance was established at *p* < 0.05.

## Results

### Data Analysis

#### Baseline Characteristics

Altogether, 315 consecutive patients were treated with the PED from July 2015 to July 2018 at our institution. Among them, 126 patients were excluded because they lacked a follow-up catheterized angiogram. Another 63 patients were excluded because no side branch involvement was evident on their angiograms. Finally, 173 involved branches in 126 patients (98 women (77.8%), 28 men (22.2%); mean age 52.9 ± 11.3 years) were included in the subsequent analyses. The demographics and clinical characteristics are shown in Table 1.

**Table 1:** Demographics and clinical characteristics.

**Characteristic**	**Results**
No. of patients	126
No. of branches covered	173
Mean age ± standard deviation (years)	52.9 ± 11.3
**SEX**	
Male	28(22.2%)
Female	98(77.8%)
Mean aneurysm size (mm)	11.2 ± 7.7
**TREATMENT STRATEGY**	
PED	88(50.9%)
PED + coil	85(49.1%)
**NO. OF PEDS**	
1	164(94.8%)
2	9(5.2%)
Mean procedural time (min)	122.4 ± 58.5
Mean length of follow-up(months)	7.9 ± 4.2
Smoking	13(7.5%)
Hypertension	66(38.2%)
Drinking	15(8.7%)
Coronary artery disease	9(5.2%)
Diabetes mellitus	7(4%)

#### Angiographic and Clinical Results

**Figure 1:**
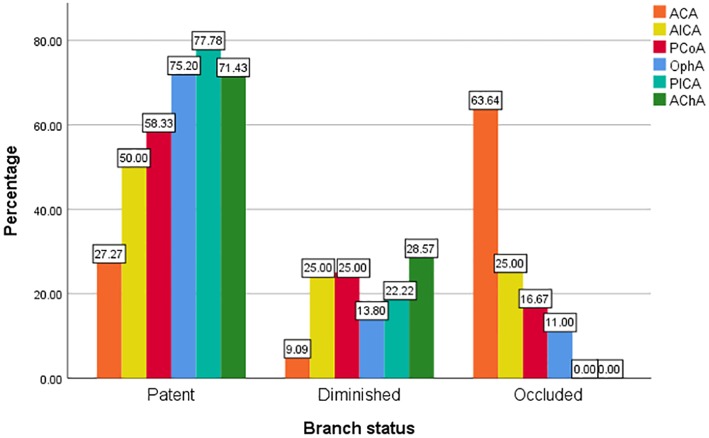
Status of the side branches involved by PED at follow-up.

The involved branches included 109 OphAs, 24 PCoAs, 7 AChAs, 11 ACAs, 4 AICAs, and 18 PICAs. Among the 24 involved PCoAs, there were 4 fetal-type posterior communicating artery. The data for the various arterial vessels regarding occlusion and diminished flow are presented in Table 2. The comparisons of status for each covered side branch at follow-up are shown in Figure 1. Aneurysms with an incorporated branch were occluded in 5 of 16 patients (31.3%), which was significantly less than those without an incorporated branch (125 of 157 patients, 79.6%; p < 0.001). Five patients with an involved OphA presented with blurred vision when awakened from general anesthesia. The symptoms disappeared several days after treatment. Follow-up angiography showed a patent OphA in all patients. One patient whose right ACA was involved and occluded immediately after the procedure complained of persistent right-sided headache. Mannitol was administered, and the patient achieved complete remission 1 week later. None of the other patients had neurologic complications or clinical strokes related to branch occlusion.

**Table 2:** Summary of branch status immediately after the procedure and at follow-up.

**Branch vessels**	**No. of vessels**	**Immediately**			**Follow-up**		
		**Patent**	**Diminished**	**Occluded**	**Patent**	**Diminished**	**Occluded**
OphA	109	104	4	1	82	15	12
PCoA	24	23	1	0	14	6	4
AChA	7	7	0	0	5	2	0
ACA	11	9	1	1	3	1	7
AICA	4	4	0	0	2	1	1
PICA	18	18	0	0	14	4	0

#### Predictors of Branch Occlusion

Univariate analysis was used to investigate the predictors of branch vessel occlusion. It revealed that a longer procedural time, smaller PED diameter, branches arising from the aneurysm, and ACA involvement were correlated with a higher occlusion rate. Significant predictors were then analyzed by binary logistic analysis, which indicated that the significant predictors of branch occlusion were a smaller PED diameter (*p* = 0.003, OR = 0.168), branches arising from the aneurysm (*p* = 0.004, OR = 6.614), and involvement of the ACA (*p* < 0.001, OR = 25.656) (Table 3).

**Table 3:** Predictors of branch occlusion.

**Variables**	**Patent branch**	**Occluded branch**	**Univariate *P*-Value**	**Multivariate *P*-Value**	**OR**	**(95% CI)**
Mean age(year)	52.8 ± 11.0	53.8 ± 13.4	0.695			
Female	116/149(77.8%)	19/24(79.1%)	0.885			
Smoking	12/149(8.1%)	1/24(4.2%)	0.800			
Hypertension	59/149(39.6%)	7/24(21.2%)	0.329			
Drinking	14/149(9.4%)	1/24(4.2%)	0.650			
Coronary artery disease	7/149(4.7%)	2/24(8.3%)	0.803			
Diabetes mellitus	5/149(3.4%)	2/24(8.3%)	0.555			
Hyperlipidemia	6/149(4%)	3/24(12.5%)	0.215			
Mean procedural time (min)	118.4 ± 57.1	147 ± 62.1	0.026	0.248	1.005	0.996–1.015
Multi PED	7/149(4.7%)	2/24(8.3%)	0.803			
Adjunctive coiling	73/149(49%)	12/24(50%)	0.927			
Mean PED diameter	4.2 ± 0.5	3.9 ± 0.4	0.002	0.003	0.168	0.052–0.540
Branch coming from aneurysm	10/149(6.7%)	6/24(25%)	0.004	0.004	6.614	1.798–24.331
Mean branch diameter	1 ± 0.5	1.2 ± 0.5	0.144			
Mean aneurysm size	11.1 ± 7.8	12.3 ± 7.6	0.481			
SAF^*^	60/149(40.3%)	9/24(37.5%)	0.797			
AOF^*^	116/149(77.9%)	15/24(62.5%)	0.104			
Balloon assisted	9/149(6%)	2/24(8.3%)	> 0.999			
Complications	5/149(3.4%)	1/24(4.2%)	> 0.999			
Initial symptoms	111/149(74.5%)	21/24(87.5%)	0.164			
Length of Follow-up	7.8 ± 4.3	8 ± 3.5	0.851			
OphA involvement	97/109(89.0%)	12/109(11.0%)	0.155			
PCoA involvement	20/24(83.3%)	4/24(16.7%)	0.914			
AChA involvement	7/7(100%)	0/24(0%)	0.599			
ACA involvement	4/11(36.4%)	7/11(63.6%)	< 0.001	< 0.001	25.656	5.271–124.875
AICA involvement	3/4(75%)	1/4(25%)	>0.999			
PICA involvement	18/18(100%)	0/24(0%)	0.150			

* *SAF, Stasis of aneurysmal flow immediately after PED deployment; AOF, Aneurysm occlusion at follow-up*.

## Discussion

Pipeline embolization devices—initially approved for treating large or giant wide-necked aneurysms between the petrous and hypophyseal segments—are being increasingly used to treat intracranial aneurysms. Recently, off-label use of PEDs for posterior-circulation aneurysms was shown to be safe and effective (9, 10). Deployment of PEDs in the anterior and posterior circulations is often accompanied by involvement of branch vessels. Although prior studies that included hemodynamic studies already proved a low occlusion rate for these involved branches and the occlusion of side branches were clinical silent (11), there are some reports of symptomatic events following branch occlusion (12). Factors that contribute to the occlusion of branch vessels are not well defined. Furthermore, most prior studies included only small numbers of samples and lacked comparisons among the various branch vessels, which therefore might not reflect the true situation. Consequently, we reviewed a large cohort of branch vessels covered by PEDs, including differences in the occlusion rates for the various branch types, patient demographics, morphologic features, procedural details, and clinical characteristics. We then collated the data to identify risk factors of branch vessel occlusion after PED deployment.

**Figure 2:**
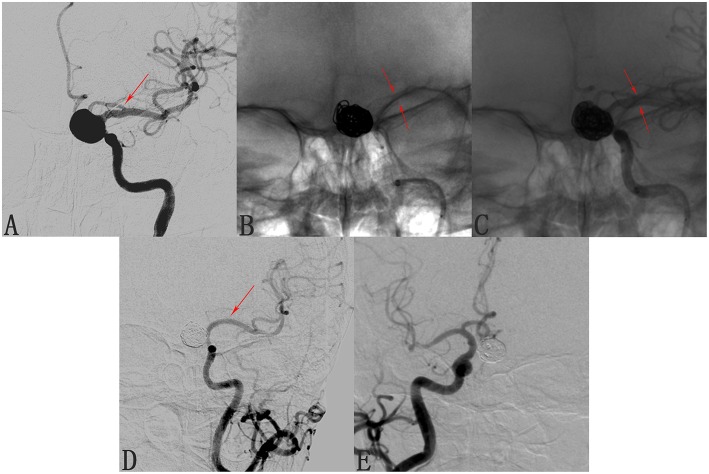
Case of an occluded anterior cerebral artery (ACA) after deploying a pipeline embolization device (PED). **(A)** Angiogram of the left internal carotid artery shows a C6 segment aneurysm and the patent ACA (arrow). **(B,C)** Angiograms shows the position of the deployed pipeline embolization device. **(D)** Follow-up angiogram 8 months after PED deployment shows compete occlusion of the aneurysm and of the left A1 segment (arrow). **(E)** Contralateral angiography shows that the left A2 segment was supplied by the anterior communicating artery, explaining the clinical silence of the ACA occlusion.

**Figure 3:**
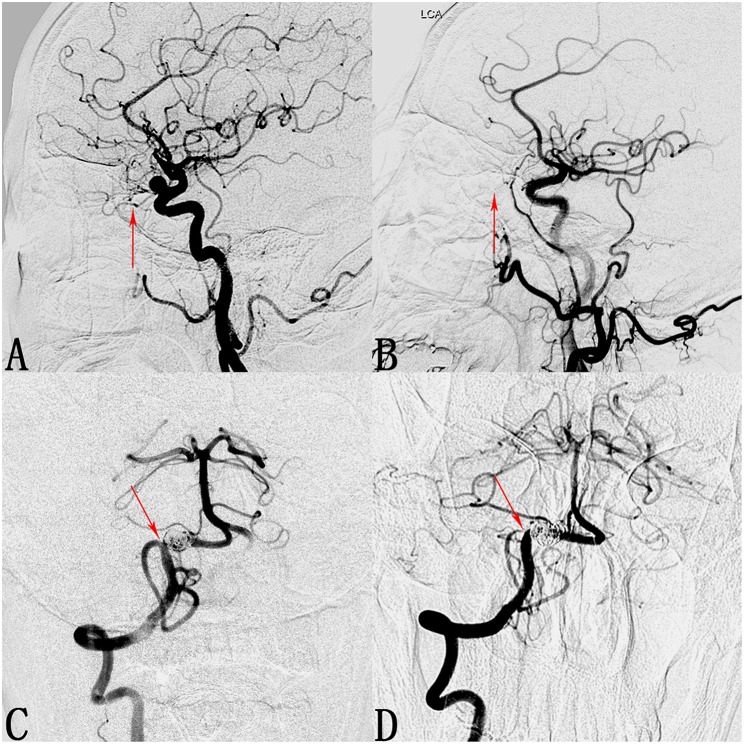
Cases of an occluded ophthalmic artery (OphA) and a posterior inferior cerebellar artery (PICA) with diminished flow. **(A)** Lateral digital subtraction angiogram shows a C6 segment aneurysm and the patent OphA (arrow). **(B)** Follow-up angiogram 3 months after PED deployment shows compete occlusion of the aneurysm and absence of the OphA (arrow). **(C)** Angiogram of the right vertebral artery shows a V4 segment aneurysm and the patent PICA (arrow). **(D)** Follow-up angiogram 10 months after PED deployment shows compete occlusion of the aneurysm and stenosis at the ostium of the PICA (arrow).

We found that 24 of 173 involved branches were occluded Figures 2, 3A,B) and 29 of 173 cases exhibited diminished flow (Figures 3C,D) at follow-up. One OphA and one A1 segment of ACA occluded immediately after PED deployment, and the rest were occluded at follow-up. Most patients with occlusions or diminished flow were asymptomatic. Figure 2 shows a representative occluded ACA (Figure 2A) at follow-up. After the ACA was covered by the PED (Figures 2B,C), despite the occlusion of A1 segment (Figure 2D), the ACA was well compensated because of the presence of a rich collateral blood supply from the anterior communicating artery (Figure 2E), thereby explaining why an occluded ACA is usually asymptomatic.

Regarding why the occlusion rate in the various side branches varies greatly, most studies suggest that it is due to the presence of a collateral supply (13, 14). OphA, PCoA, and ACA have a rich collateral supply from the external carotid artery, posterior cerebral artery, and anterior communicating artery, respectively. When covered by the PED, branches with rich collateral supply have a lower pressure gradient and are more inclined to occlude than branches without a collateral supply. Side branches such as the AChA, AICA, and PICA, which do not have a collateral supply, have a high patency rate after PED deployment. This theory could well explain the differences in the occlusion rates in the various branches.

ACA involvement was associated with the highest occlusion rate (63.64%) and proved to be a significant predictor (*p* < 0.001, OR = 25.656) of branch occlusion according to the multivariate analysis. We also found that a smaller PED diameter was associated with branch occlusion (*p* = 0.003, OR = 0.168), which had also been reported by Miller et al. (15). They believed that smaller PEDs have greater metal density than larger devices and thus exert a higher blocking effect on the flow in branch arteries. In addition, our results revealed that branches arising from the aneurysm are risk factors for branch occlusion (*p* = 0.004, OR = 6.614). We supposed that, when involved with an aneurysm, branch arteries were more inclined to be occluded along with it. The inner mechanism of this tendency has not yet been well defined, however, and requires further hemodynamic and clinical study.

The procedural time (*p* = 0.248, OR = 1.005) might be another risk factor for branch occlusion. In the present study, the mean procedural time was 118.4 ± 57.1 min for the patent group and 147 ± 62.1 min for the occluded group. Despite its lack of significance in the binary logistic regression analysis, we believe that minimizing the procedural time is beneficial for the patency of branches after PED deployment.

Prior studies have investigated the flow alteration of side branches after PED deployment using computational fluid dynamics (CFD). Hu et al. (16) studied 31 patient-specific AICAs covered by virtually implanted PEDs using CFD. The blood flow reduction ratio was shown to be 3.6 ± 1.9%. They found that a larger branch diameter was correlated with a higher blood flow reduction ratio, which is consistent with the high occlusion rate of the ACA. Their research showed that PED had little blocking effect on flow into the involved branches. *In vivo* studies of branches covered by PED were conducted to examine the patency and local histologic alteration after PED deployment. Experiments in rabbits (17) and miniature pigs (18) confirmed the patency of branch vessels at follow-up. Neointima consisting of collagenous fibers, smooth muscle cells, and macrophages partially covered the ostia of the branch vessels, but the lumens at the ostia were still patent in all cases. Thicker neointima was measured in cases of two and three PEDs. Similar results have been reported on the patency of OphA (14, 19, 20), PCoA (8, 21), AchA (22–24), and posterior circulation branches (13).

Although branches covered by the PED are usually patent and asymptomatic, there are some reports of branch occlusion and related symptoms. Lall et al. (12) reported three cases of acute branch occlusion after PED deployment, even though they were pretreated with a dual antiplatelet regimen. Immediately after awakening from general anesthesia, the patient in case 2 was having difficulty following commands, had dysarthric speech, and exhibited lower cranial neuropathy. Emergency angiography showed occlusion of the left PICA. Phillips et al. (25) reported three cases of perforator territory infarction after PED treatment of posterior circulation aneurysms. Hence, when choosing treatment strategies, neurointerventionalists should avoid coverage of branch arteries (especially the ACA), if possible. In addition, minimizing the procedural time and choosing larger-diameter PEDs are valid strategies for lowering the occlusion rate.

Our study has several limitations. First, because of the limited number of occluded cases, the risk factors for branch occlusion might not have been well revealed. Second, because some branches were found to have developed stenosis at follow-up, these branches may be found to have become occluded at a longer follow-up. Thus, the short follow-up time may reduce the accuracy of our results. Longer follow-up results are needed in future studies.

## Conclusions

The results of our study suggested a low rate of branch occlusion after PED deployment. Branches with a rich collateral supply are more inclined to become occluded after PED deployment—e.g., involvement of the ACA was associated with the highest occlusion rate. A smaller PED diameter, branches arising from the aneurysm, and involvement of the ACA were significant predictors of branch occlusion. To our knowledge, this is the first study to report that branches arising from the aneurysm and ACA involvement are predictors of branch occlusion after PED deployment.

## Data Availability

The datasets generated for this study are available on request to the corresponding author.

## Author Contributions

SM, XY, and YZ contributed to conception and design of the study. XW, ZT, and YYZ collected the data. XW and WL performed the statistical analysis. XW wrote the first draft of the manuscript. JL and YSZ wrote sections of the manuscript. All authors contributed to manuscript revision, and read and approved the submitted version.

### Conflict of Interest Statement

The authors declare that the research was conducted in the absence of any commercial or financial relationships that could be construed as a potential conflict of interest.
